# Catheter-Induced Thrombosis of the Superior Vena Cava

**DOI:** 10.1155/2012/469619

**Published:** 2012-11-06

**Authors:** Elio Venturini, Lucia Becuzzi, Lucia Magni

**Affiliations:** Department of Cardiology, Civic Hospital, 57023 Cecina, Italy

## Abstract

There has been an increase in the use of central venous catheters (CVCs) in clinical practice. One of the most dangerous complications associated with their use is symptomatic or asymptomatic thrombosis (T), sometimes associated with superior vena cava (SVC) syndrome, resulting from impaired venous drainage. The right heart clots can induce an increased risk of mortality due the potential pulmonary embolism (PE). We report a case of asymptomatic 83-year-old woman in whom the thrombosis was detected after an echocardiogram. Echocardiography demonstrated a cardiac mass, and the T was confirmed by (magnetic resonance imaging) MRI. The clinical scenario, a high index of suspicion and routine use of echocardiogram in patients with CVC, can lead to a correct diagnosis, preventing dangerous complications.

## 1. Introduction

 The CVCs are widely used both for the parenteral nutrition that for chemotherapy or dialysis. They are easy to implant under local anesthesia and reduce the patient's discomfort by administering drugs otherwise harmful to the peripheral veins. However there are possible early or late complications related to implantation technique, care, or maintenance. 

 The syndrome of SVC, stenosis or occlusion of the SVC, with venous outflow obstruction of the head and upper extremities, is rarely induced by CVC. Usually it is diagnosed in the setting of malignancy (60–85%). Mediastinal fibrosis, indwelling CVC, or pacemakers wires are the cause of the rest of (40–15%) benign cases of the syndrome [1].

 The risk of clot depends on many variables such as the patient, the technique used, the site of puncture, the type of catheter, and the liquid infused. However data on CVC-related T are inconsistent. The true incidence may be underestimated because many patients are asymptomatic. Even in the presence of symptoms the diagnosis is misunderstood. In indwelling venous devices varies from 1.5 to 13% [2], while in the central catheters inserted from peripheral vein it is between 2 and 4% [3].

 We describe the case of an 83-year-old woman with a SVC T extended to right heart chambers, highly mobile and prolapsing through the tricuspid valve.

## 2. Case Report

 The patient was referred by physiatrists for dyspnea in suspected PE. She had a previous hypertension and results of stroke with left hemiparesis. The previous month she underwent surgery for peritonitis from perforation of the duodenum with subsequent hospitalization in intensive care. Bemiparin (3.500 U.I/die) was used as thromboembolism prophylaxis. She was treated with total parenteral nutrition, and the CVC (left subclavian vein) was maintained for 15 days and then removed.

The patient showed a general weight loss and wasting for long-term hospitalization. However, during physical examination, a normal arterial blood pressure of 140/80 mmHg, regular and rhythmic cardiac activity without murmurs, abnormal sounds, and no signs of heart failure were detected. The ECG was normal.

**Figure 1:**
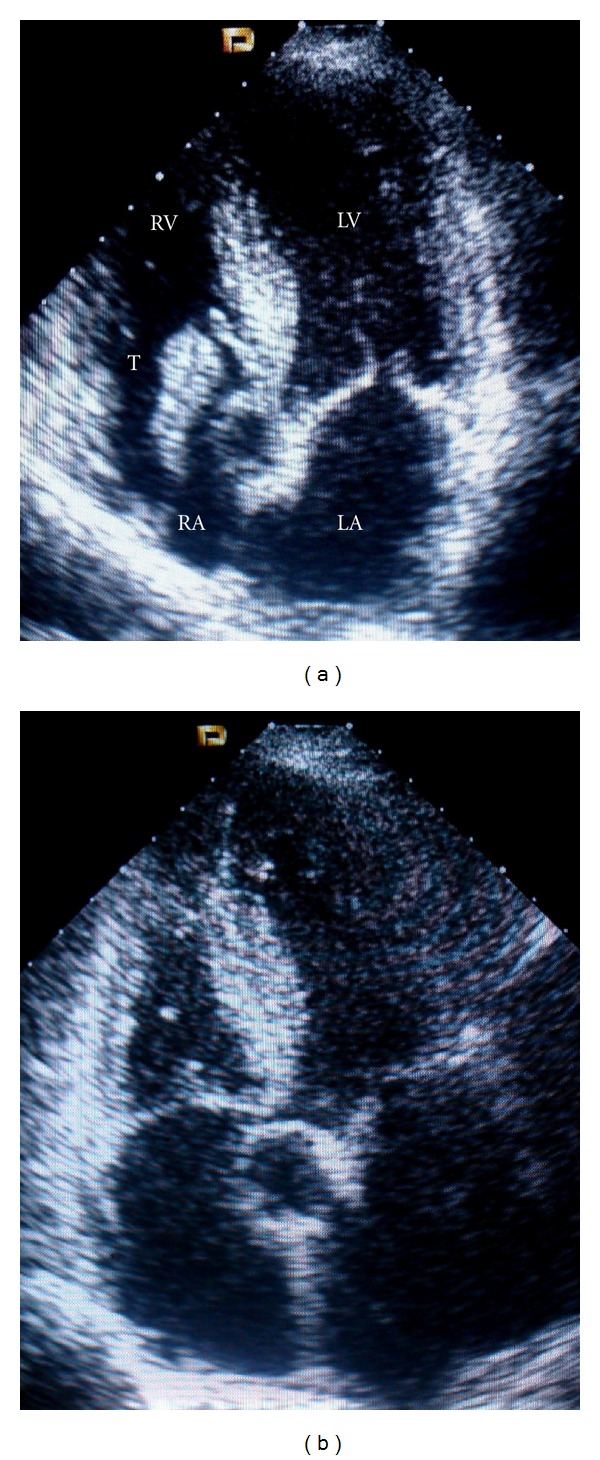
(a) Transthoracic apical five-chamber view showing the thrombus with thin stalk prolapsing through the tricuspid valve. (b) Same view after oral anticoagulation therapy. Complete dissolution of the thrombus. LA, left atrium; LV, left ventricle; RA, right atrium; RV, right ventricle; T, thrombus.

 During transthoracic echocardiography a mass in the right atrium from the SVC with a thin stalk, mobile was noted. This undertook the tricuspid valve during diastole (Figure 1(a)).

 The Doppler ultrasonography of the upper extremities also showed the T of the veins, internal jugular, subclavian, and axillary right.

 Contrast-enhanced MRI demonstrated homogeneous hyperintensity compared to the myocardium on images black-blood DP-weighted and T2-weighted, with maximum dimensions, 10 × 8 mm, originating from the left brachiocephalic vein (Figure 3). The mass extended caudally into the right atrium, fluctuating between venous and muscular portions of the atrium and engaging through the tricuspid valve into the right ventricle (Figure 4).

**Figure 2:**
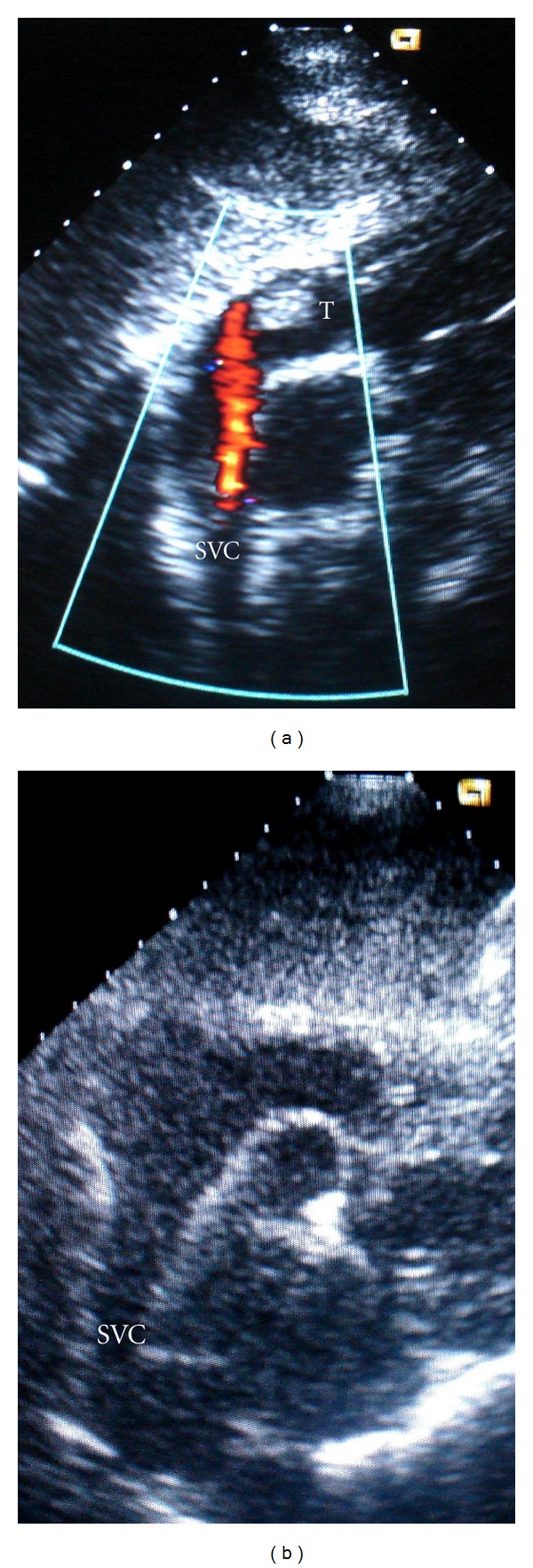
(a) Subcostal short axis projection. Thrombus in the SVC. Incomplete obstruction as shown by the flow approaching the probe. (b) Same view after oral anticoagulation therapy. SVC: superior vena cava.

**Figure 3:**
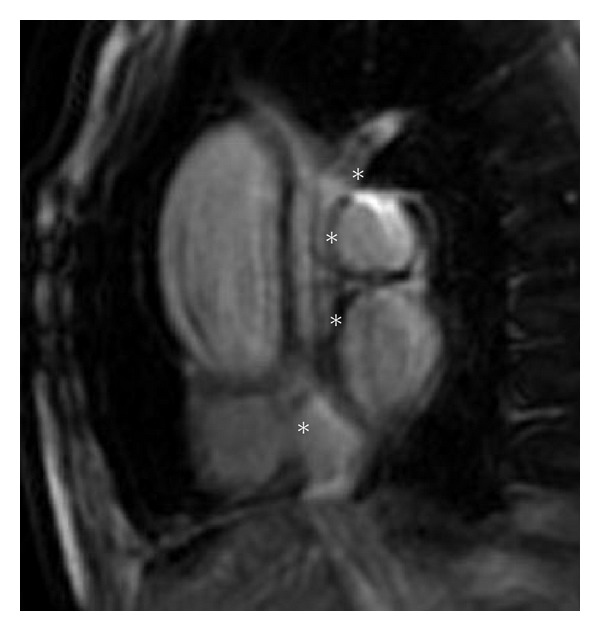
MRI showing the entire course of the thrombus (*) from the left brachiocephalic vein, SVC, and finally to the right atrium.

**Figure 4:**
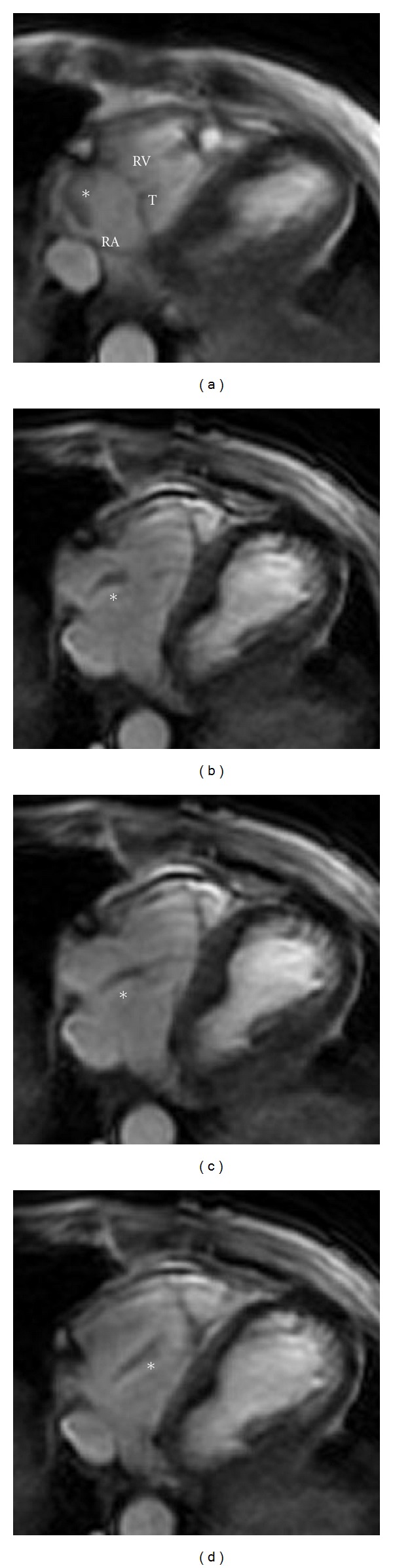
MRI. Sequence of the movement of the thrombus (*) in the right heart. During the systole is “rolled” in RA (a), in isovolumic diastole “lengthens” ((b), (c)) to then prolapse in RV, in end-diastole (d).

 A CVC-induced T was diagnosed. Given the high surgical risk and the possibility of PE, the patient was treated conservatively, first with enoxaparin (6.000 U.I. bid) and then, with warfarin target INR 2-3. After 3 months of therapy the T was completely resolved (Figures 1(b) and 2(b)).

 At 12-month followup the patient is in good conditions with no recurrences of the disease.

## 3. Discussion

 The T, CVC-induced, recognizes several causes. The vein can be damaged by hyperosmolar fluid or at the time of insertion of the catheter and stasis can be induced by its presence. Moreover the repeated trauma of the tip of catheter, induced by cardiac activity, can injure the endothelium surface [4]. The hypercoagulable state as that of cancer, myeloproliferative diseases [5], trauma, or surgery (as in the described case) must be considered a predisposing factor. 

 All these factors are the *Virchow's triad*. Furthermore the risk of thrombosis is related to the permanence of CVC and is higher in elderly and systemically ill patients [6].

 Not rarely the SVC T is asymptomatic; symptoms, when present, are usually upper limbs edema and a slight shoulder and neck pain [7]. The complete vein occlusion is associated with the classic *SVC syndrome*: arm and facial swelling, stridor, blurred vision, dyspnea, dizziness, positional headache, retroorbital pain, dysphagia, and chest pain [6]. The chronic and inveterate obstructions can be associated with the signs of increased cervical venous pressure.

 Such complications are normally observed while the catheter is in place. In our patient the thrombosis was demonstrated after the removal of the CVC; we found only one similar clinical case reported [8]. In addition, the T did not induce complete occlusion of the SVC, (as is clearly shown in Figure 2) and this explains why the patient was essentially asymptomatic.

 Since the clinical presentation is often silent or nonspecific, the diagnosis can be incidental by duplex ultrasound, echocardiography, CT, or MRI. In our patient the echocardiography was fundamental for the diagnosis of cardiac mass. For the differential diagnosis, the myxoma, though pedunculated and mobile, is usually left atrial sided rather than right sided.

 The proof of a thrombotic occlusion of the veins by Doppler ultrasonography of the upper extremities increases the diagnostic suspect finally confirmed by CT or MRI.

 These techniques have a high diagnostic accuracy with excellent anatomic definition of the whole T and its relationship with adjacent structures. Moreover they show the collateral pathways. However, the use of ionizing radiation for the CT and of contrast agents with the risk of nephrotoxicity makes them unsuitable in some patients [4].

 Oral anticoagulation is the first choice therapy. Surgical thrombectomy and thrombolysis are indicated in cases of massive T and hemodynamic instability and PE [3, 5]. Balloon angioplasty and stent placement can be useful in the case of chronic obstructions and SVC syndrome [6].

 For the elderly, the poor general condition, the incomplete vessel thrombosis, the absence of PE, but, on the other hand, the risk of clot migration after stalk lysis, the patient was treated with warfarin at target INR 2-3, with good results.

 The CVC-induced T is a challenge. The clinical presentation, often silent, may lead to underestimation. Only a high index of suspicion may allow early detection, before the onset of complications.

 The patient's general condition (cancer, multiple trauma, major surgery, systemic diseases, cachexia, and dialysis), difficult insertion of CVC, particularly if followed by thrombocytopenia, [3] should prompt the suspicion of T CVC induced.

 The optimal management and care of CVC must provide a routine duplex ultrasound and echocardiogram of the upper limbs veins, of the SVC, and of the right heart's chambers. This will avoid complications even lethal.
